# Associations of Lumber Disc Degeneration With Paraspinal Muscles Myosteatosis in Discogenic Low Back Pain

**DOI:** 10.3389/fendo.2022.891088

**Published:** 2022-05-13

**Authors:** Yilong Huang, Ling Wang, Baofa Luo, Kaiwen Yang, Xiaomin Zeng, Jiaxin Chen, Zhenguang Zhang, Yanlin Li, Xiaoguang Cheng, Bo He

**Affiliations:** ^1^ Department of Medical Imaging, The First Affiliated Hospital of Kunming Medical University, Kunming, China; ^2^ Department of Radiology, Beijing Jishuitan Hospital, Beijing, China; ^3^ Department of Sports Medicine, The First Affiliated Hospital of Kunming Medical University, Kunming, China

**Keywords:** discogenic low back pain, quantitative MRI, paraspinal muscles, fatty infiltration, lumbar intervertebral disk degeneration, inflammation

## Abstract

Accompanied with intervertebral disc (IVD) degeneration, increasing fat infiltration of paraspinal muscles may be related to discogenic low back pain (DLBP), but their relationship is still unclear and the classical animal models are not completely applicable. The purpose of this study was to assess the paraspinal muscle fat infiltration in patients with DLBP by quantitative MRI, and to develop a novel DLBP rat model to explore the potential relationship between DLBP paraspinal muscle fat infiltration and TNF-α levels. We measured the proton density fat fraction (PDFF) of the multifidus and erector spinae muscles of 70 DLBP patients and 36 healthy volunteers by using quantitative MRI IDEAL-IQ. In addition, we developed a DLBP experimental rat model by puncturing the L4/5 and L5/6 IVDs under the guidance of X-ray fluoroscopy. Then various behavioral experiments, MRI and pathological examination of IVDs were used to evaluate the performance of the DLBP animal model. The gait analysis, hot plate test, acetone test, grasping test and tail suspension test were used to evaluate the pain and muscle dysfunction in rats. Through quantitative MRI and histological examination, the degeneration of IVDs and fat infiltration in the muscles were observed *in vivo* and ex vivo. Enzyme linked immunosorbent assay detects the level of TNF-α in rat IVDs and paraspinal muscles. In the human study, compared with healthy volunteers, the PDFF of multifidus and erector muscles of DLBP patients increased significantly at L4/5 and L5/S1 levels (p<0.05). In the rat experiment, compared with control group and sham group, DLBP group had reduced gait score, shortened response time to cold and heat stimuli, prolonged bending time, and shortened struggling time. Rat lumbar MRI T2WI showed that the signal intensity of L4/5 and L5/6 IVDs were progressively decreased. Histological examination revealed that IVDs had increased collagen fibers, reduced nucleus pulposus, thickened annulus fibrosus, and distorted shape. The PDFF of multifidus muscle at L4/5 and L5/6 level in the DLBP group were more than that in other groups (p<0.05), and HE staining and oil red O staining of paraspinal muscles showed that the muscle bundle space of the DLBP group muscles increased, and the muscle tissues Increased lipid droplets. Finally, the expression of TNF-α in IVDs and paraspinal muscles in the DLBP group were significantly higher than that in the control group (p<0.05). It is reliable and feasible to establish a DLBP rat model by puncturing the lumbar IVDs under the guidance of X-ray fluoroscopy. The degeneration of lumbar IVDs with DLBP leads to the occurrence of fat infiltration of paraspinal muscles, which is related to the expression of TNF-α.

## Introduction

Low back pain (LBP) is one of the most important challenges to global public health which leads to great economic burdens on society (1, 2). Discogenic low back pain (DLBP) is the most common type of chronic LBP, accounting for 39% of patients with chronic LBP (3). Intervertebral disc (IVD) degeneration is considered to be the leading cause of DLBP especially related to inflammation (4), but treatment is mainly limited to partial symptom relief (5). Paraspinal muscles (multifidus, erector spinae, and psoas major) are crucial to the stability and function of the lumbar spine (6). Muscle atrophy and fat replacement are considered to be the features of DLBP paraspinal muscle remodeling, and fat infiltration may worsen DLBP (7). Given the meaningful role of paraspinal muscles on the lumbar spine, fat infiltration may worsen DLBP. Therefore, it’s crucial to assess the interaction between the fat infiltration in the paraspinal muscles and DLBP.

Previous studies have reported decreased computed tomography attenuation (8, 9) and increased T2-weighted imaging (T2WI) signal (10) in the paraspinal muscles of LBP patients due to fat infiltration. In recent years, the advanced chemical shift-encoded MRI (CSE-MRI) has been proposed for non-invasive quantitative assessment of fat in various parts of the human body (11, 12). The proton density fat fraction (PDFF) estimated by using CSE MRI is highly repeatable and accurate in the quantitative assessment of muscle fat composition (13). The iterative decomposition of water and fat with echo asymmetry and least-squares estimation (IDEAL-IQ) is one of CSE-MRI techniques. IDEAL-IQ is considered to be comparable to MR spectroscopy (the gold standard method *in vivo*) for quantification of muscle fat infiltration (14, 15). Further, IDEAL-IQ or other CSE-MRI imagings have significant advantages over magnetic resonance spectroscopy, such as rapid and volumetric data acquisition with visualization of anatomical structures. Therefore, PDFF may help to clarify and quantify the fat infiltration in the paraspinal muscles of DLBP.

In clinical studies, the muscle fat infiltration of patients is affected by many factors, such as age, gender, BMI, exercise etc (16). The direct causal relationship between IVD degeneration and paraspinal muscle fatty infiltration is unclear. Therefore, the study of animal experiments with single variable control is very necessary. In animal experiments, previous studies have proposed several *in-vivo* animal DLBP models (17–20), but none of them seem to be suitable for this study. Current *in-vivo* models can be categorized into natural models and artificial models. The natural model was age-related long-term IVDs degradation, just like humans. However, such models take a long time to implement and have the shortcoming of individual symptom variation and high cost. The artificial model aims to destroy the IVDs, leading to common clinical manifestations of DLBP similar to humans in animals. This method has been widely adopted for its simplicity and high efficiency. Several studies have demonstrated the possibility of surgically incising the abdomen or back and accurately destroying the IVDs to obtain effective DLBP animal models (18–21). But these DLBP animal models also destroy the muscles, nerves and bone structures around the spine. In contrast to the previous animal models, the animal IVDs are punctured under the guidance of X-ray fluoroscopy, which can accurately destroy the IVDs and ensure that other important structures of the lumbar spine are not damaged and integrity, especially the paraspinal muscles. This approach avoids paraspinal muscles being disturbed by surgery whenever possible, which is beneficial to further investigate the relationship between DLBP and paraspinal muscles. Besides, this animal model is more efficient, economical and may more closely resembled the process of natural IVDs degeneration.

In previous studies, inflammatory factors are an important player in IVD degeneration, especially TNF-α, and chronic low-level TNF-α may be an important factor in promoting the white adipogenesis of skeletal muscle (22–24). In summary, we propose the hypothesis that in DLBP patients and new animal models, the IVDs degeneration leads to fat infiltration of the paraspinal muscles, which is associated with inflammation.

In this study, firstly, we aim to clear the phenomenon of paraspinal muscle fat infiltration in DLBP patients by accurate quantitative MRI (IDEAL-IQ technology). Then, after establishing and evaluating a new DLBP animal model, we further revealed the direct causal relationship between DLBP and paraspinal muscle fatty infiltration, and explored the potential relationship between fatty infiltration and inflammatory response.

## Materials and Methods

### Participants

Seventy patients with DLBP and thirty-six healthy volunteers were recruited in this study from January 2019 to May 2021 (53 males, 53 females; mean age: 44.50 years; age range: 21-74 years). Informed consent forms were signed by each participant, and ethical committee approval was obtained. The inclusion criteria were as follows: the untreated patient has symptoms of LBP for more than 3 months; Healthy volunteers have no symptoms of LBP; BMI ranged from 18.7 to 23.4 kg/m^2^. The exclusion criteria were visceral LBP (such as urinary tract stones); lumbar disc herniation, spinal stenosis, spinal trauma, fracture, tumor, infection, deformity, spondylolisthesis, surgery and other musculoskeletal diseases; pregnancy; and contraindications for MRI.

### Animals

Male Sprague−Dawley (SD) rats (200−250 g) were used as experimental animals. The rats were raised under specific pathogen-free conditions and kept the ambient temperature (24-26°C) and humidity (30-50%) constant. Sterilized food and water were provided to all rats. All animal experiments were approved by the Institutional Animal Care and Use Committee of Kunming Medical University (approval number: Kmmu2021038).

### DLBP Rat Model

**Figure 1:**
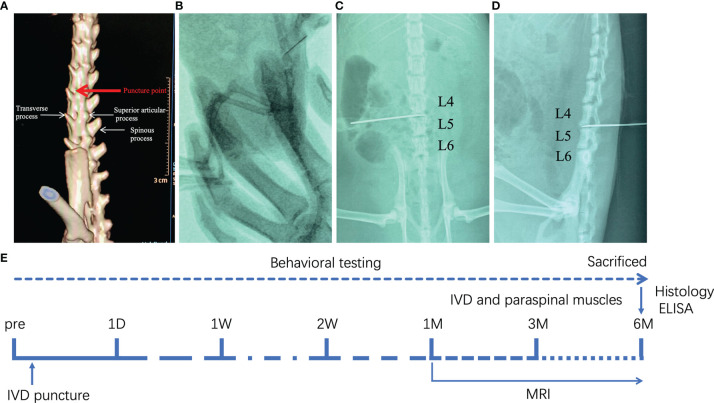
Establishment of DLBP rat model guided by X-ray fluoroscopy. **(A)** The location of the needle puncture; **(B)** X-ray fluoroscopy; **(C, D)** X-ray lumbar spine positive and lateral positioning needle position in the IVD of L4/5 and L5/6; **(E)** the experimental schedule.

We randomly divided sixty SD rats into three groups: control group, sham group and DLBP group (n = 20, each group). The rats in the DLBP group were anesthetized with an intraperitoneal injection of sodium pentobarbital (40 mg/kg). The rat was placed in the right decubitus position. Under the guidance of X-ray fluoroscopy, a Hamilton syringe (27G) was slowly inserted into the L4/5 and L5/6 IVDs from the posterior of the back. The puncture point was located between the transverse process and superior articular process, and the angle between the needle and horizontal plane was 45°. Anteroposterior and lateral X-ray examination of lumbar spine was used to confirmed that the needle was located in the central area of ​​IVD (**Figures 1A–D**). The nucleus pulposus was destroyed through the needle, and sterile PBS (2.5 μL) was injected. The fluoroscopy conditions were tube voltage 52 kV and tube current 0.6 mAs. The sham group only punctured the paraspinal muscles at the L4/5 and L5/6 levels but did not puncture the IVDs. To exclude the interference caused by the puncture of paraspinal muscle, the sham group was set up. No operation in the control group. After the operation, the rats were placed under a warm lamp, put back into home cages, and recovered from anesthesia.

### Behavioral Tests

#### Gait Analysis

At 1 day, 1 week, 2 weeks, 1 month, 3 months, and 6 months after surgery, behavioral tests were performed (gait analysis, hot plate test, acetone test, grasping test and tail suspension test) to evaluate the pain and dysfunction of the low back in the rats (**Figure 1E**).

First, the three groups of rats were scored for gait disorder (25). The rat was placed on the experiment table and its walking performance was observed. Scoring was based on the following criteria: 3 = severe dyskinesia and unable to walk; 2 = moderate dyskinesia, able to walk, but obvious limp; 1 = mild limp; 0 = normal physical activity.

#### Hot Plate Test

Hot plate test was used to evaluate the pain threshold to thermal stimuli (26). Rats were allowed to walk on the hot plate (55.0 ± 0.5°C) for up to 20 s. The time between the placement of the rats on the hot plate and licking the paws or jumping was recorded as the response time. Hot plate test was performed two times per rat with an intervening interval of 10 minutes.

#### Acetone Test

Cold hypersensitivity was assessed *via* the acetone test as previously described (27).

The acetone (50 μL) in the syringe was gently touched to the plantar skin of the left and right hind paw of the rat. Within 1 minute, the time from skin contact with acetone to paw flinching and biting was recorded. The average time on the left and right paw was taken as the response time.

#### Grasping Test

Grasping test was used to evaluate myodynamia (28). Briefly, the limbs grasped the upside-down iron net until it was unable to hold on within 20s. The time of grasping on the iron net reflects the muscle strength of the rat. Grasping test was measured twice and the mean value was calculated.

#### Tail Suspension Test

Tail suspension test (TST) was performed to evaluate muscle function in a specially manufactured tail suspension box (29). The rat was suspended 50 cm above the bottom of box by fixing its tail tip (1 cm) with adhesive tape. The time the rats struggled and bent over within 5 minutes was recorded respectively.

### MR Data Acquisition

All MRI experiments were performed using a 3.0T MR system (Discovery 750w, GE Healthcare, USA). A 32-channel phased array spine coil was used for DLBP patients and healthy volunteers. To reduce motion artifacts, an abdominal bandage was used to compress the abdomen and a wedge-shaped foam pad was placed under the lower limbs of participants in a standard supine position. MRI scanning for participants included sagittal T1-weighted imaging (T1WI), T2WI of lumbar spine and axial T2WI, IDEAL-IQ of paraspinal muscles. A dedicated animal coil with an inner diameter 70 mm (CG-MUC49-H300-AG, Chenguang Medical Technologies, China) was applied for rats. After being anesthetized by an intraperitoneal injection of pentobarbital sodium (45 mg/kg), the rat was placed in a standard prone position. The towel maintains the rat’s body temperature. Animal MRI included sagittal T2WI and IDEAL-IQ. The MRI protocols of participants and rats are summarized in **Table 1**.

**Table 1:** MRI scan parameters.

Images		TE (ms)	TR (ms)	ST (mm)	SL (mm)	FOV (mm^2^)	NEX
Participants							
Spine	Sagittal T1WI	8.3	361	4	4	320×180	2
	Sagittal T2WI	142	2500	4	4	320×180	2
Muscles	Axial T2WI	110.5	4024	3	4	20×20	3
	IDEAL-IQ	1.2/3.2/5.2/ 7.2/9.2/11.2	7.8	4	0	24×24	2
Rat							
Spine	Sagittal T2WI	100	3190	1.2	0	10×10	4
Muscles	IDEAL-IQ	Min Full	14.4	2.0	0	15×15	2

T2WI, T2-weighted imaging; TR, time of repetition; TE, echo time; ST, slice thickness; SL, slice increment; FOV, field of view; NEX, number of excitation.

### Image Analyses

**Figure 2:**
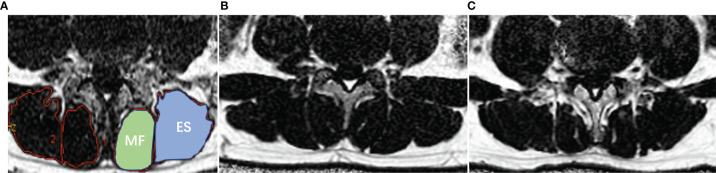
Demonstration of paraspinal muscle segmentation and MRI PDFF of lumbar paraspinal muscle in patients with DLBP. **(A)** Processed PDFF maps of paraspinal muscles; manual segmentation of paraspinal muscles. MF: multifidus, and ES: erector spinae. **(B)** Healthy Volunteer, male, 32 years old, PDFF _left MF_ = 7.3%, PDFF _right MF_ = 7.8%, PDFF _left ES_ = 8.5%, PDFF _right ES_ = 8.7%. **(C)** Patient with DLBP, male, 28 years old, DLBP for 6 years, PDFF _left MF_ = 18.4%, PDFF _right MF_ =19.7%, PDFF _left ES_ =12.0%, PDFF _right ES_ = 13.9%.

All raw MR images were transferred to a commercially available workstation (Advantage Windows 4.6, GE Medical Systems, USA). Proton density fat fraction (PDFF) was automatically postprocessed with a vendor-provided algorithm after MRI scanning. PDFF values of the bilateral paraspinal muscles were obtained on a region of interest (ROI) basis at the central level of human L4/5 and L5/S1(**Figure 2A**), and rat L4/5 and L5/6. The edges of ROI were closed to the epimysiums of multifidus and erector spinae, excluding the subcutaneous fat and fat under profundal fascia. The two radiologists manually delineated the shape of the bilateral multifidus and erector spinae. The relative signal intensity of rat IVD = T2 signal intensity _L4/5 or L5/6_/T2 signal intensity _L3/4_. The average of the two measurements was calculated and used for later analysis. ICC Coefficients showed a high reliability of estimates between two radiologists (ICC = 0.939, 95%CI: 0.852-0.975, p<0.001).

### Histopathological Analysis

Rats in the three groups were sacrificed after the last MRI acquisition. After perfusion, the lumbar spine and paraspinal muscles at levels L4/5 and L5/6 were post-fixed in 4% paraformaldehyde. After immersion in decalcification solution for two days, the vertebral tissues were dehydrated. The decalcified lumbar spine and muscles were infiltrated and embedded with paraffin to form paraffin blocks. Then, the IVD and muscles were sliced into 5-mm-thick sections. Slices from the frontal plane were treated with xylene to remove paraffin, rehydrated in a gradient alcohol bath, and rinsed three times with PBS. The sections were respectively stained with HE and safranin O/fast green staining. Part of the separated paraspinal muscles were frozen with liquid nitrogen, and 10-micron frozen sections were obtained at -20°C. The sections were used for oil red O staining later (30). Histological images were obtained by a digital microscope.

### Evaluation of TNF-α Levels

The protein levels of the cytokine TNF-α of IVDs and paraspinal muscles were measured in the rats by enzyme linked immunosorbent assay (ELISA). The IVDs and paraspinal muscles samples of rats were collected and weighed. The tissues were ground using a mortar and pestle, and homogenated in the PBS solution. High-speed hypothermal centrifugation (12000 rpm, 4°C) was performed for 10 min and the supernatant was obtained. The TNF-α protein levels were detected using an ELISA test as previously described (31). Briefly, the cytokine TNF-α protein levels were quantified using a commercially available rat-specific ELISA kit (Rat TNF-α, ml002859, Meilian, Shanghai, China) according to the manufacturer’s instructions. The samples were read at 450 nm using a spectrophotometer (SpectraMax, MD, USA).

### Statistical Analysis

SPSS 22.0 was performed for the statistical analysis. Normality was tested with the Shapiro–Wilk normality test. Mean ± SD was used to express data. Comparisons between patients with DLBP and healthy volunteers were determined using the independent-sample *t*-test. One-way analysis of variance (ANOVA) was employed for the comparisons among multiple rat groups, and Tukey’s multiple comparisons test was utilized for the *post hoc* test after ANOVA. A *p-*value <0.05 was reported statistically significant.

## Result

### Comparison of PDFF in the Paraspinal Muscle Between Healthy Volunteers and Patients With DLBP

**Table 2** shows the baseline clinical characteristics of the participants. The PDFF of the multifidus and erector spinae of different levels of DLBP were significantly higher than those of healthy volunteers in the multifidus and erector spinae at L4/5 and L5/S1 level, and the differences were statistically significant (P<0.05, **Table 2**). The PDFF of paraspinal muscles were elevated in 1.79 times in patients with DLBP as compared to control (15.05 ± 4.50% *vs.* 8.43 ± 3.56%). **Figures 2B, C** shows the PDFF maps of the paraspinal muscles of healthy volunteers and DLBP patients. Patients with DLBP have increased fat infiltration of paraspinal muscles.

**Table 2:** Comparison of Baseline Clinical Characteristics and PDFF of Paraspinal Muscle between Healthy Volunteers and DLBP Patients.

Characteristics	Healthy volunteers (n=36)	DLBP (n=70)	*p*
Male/Female	18/18	35/35	1.000
Age (year)	47.57 ± 15.78	41.67 ± 14.01	0.182
BMI (kg/m^2^)	21.97 ± 1.60	22.57 ± 1.59	0.063
PDFF of MF (%)			
L4-5	7.49 ± 2.1	13.88 ± 4.70	<0.001^*^
L5-S1	8.03 ± 3.78	14.51 ± 4.24	<0.001^*^
PDFF of ES (%)			
L4-5	7.97 ± 2.8	14.57 ± 3.36	<0.001^*^
L5-S1	10.21 ± 4.63	17.17 ± 5.05	<0.001^*^

DLBP, Discogenic low back pain; PDFF, proton density fat fraction; MF, multifidus; ES, erector spinae. All values were expressed as mean ± standard deviation. Significant p-values are marked with “*”.

### Analysis of Pain and Dysfunction Behavior in the DLBP Rats

**Figure 3:**
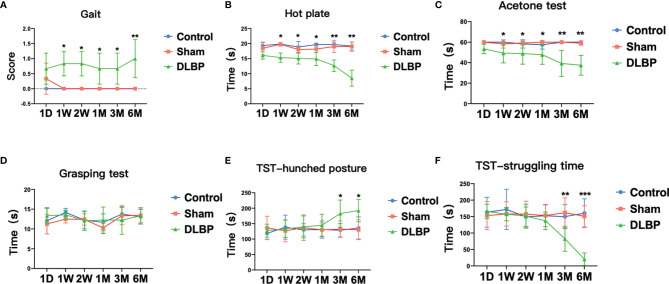
Pain and dysfunction behavior in rats after intervertebral disc puncture. **(A)** Gait analysis; **(B)** Hot plate test, which assess thermal sensitivity; **(C)** Acetone test, which assess cold allodynia. **(D)** Grasping test; **(E, F)** TST, Tail suspension test, hunched posture **(E)** and struggling time **(F)**. Compared with control group, *p < 0.05, **p < 0.01, ***p < 0.001.

After puncturing the lumbar IVD under X-ray guidance, the rats’ gait, pain threshold to thermal and cold stimuli, grip strength and lumbar muscle function were observed. First, the gait score of the DLBP group was higher than that of the sham group and normal control group from the 2 weeks to the 6 months, and the difference was statistically significant (mean 0.78 ± 0.54 *vs.* 0.06 ± 0.23 *vs.* 0.00 ± 0.00, P <0.05, **Figure 3A**).

On the 1 week after the operation, compared with the normal group and the sham group, the DLBP group rats had increased sensitivity to heat and cold stimulation, and shorter tolerance latency in the hot plate test and the acetone test (P <0.05, **Figures 3B, C**). Compared with the normal group, the tolerance latency of the hot plate test in the DLBP group was shortened by 4.50s, 3.70s, 4.70s, 7.00s and 10.67s from 1W to 6M, respectively. The tolerance latency of the acetone test in the DLBP group was shortened by 10.67s, 9.42s, 9.75s, 20.75s and 22.50s from 1W to 6M, respectively. However, the difference in grip strength among the three groups was not statistically significant (P>0.05, **Figure 3D**). In addition, the rats in the DLBP group had longer bending time and shorter struggling time than the normal group and sham group in the TST in the 3M and 6M (P<0.05, **Figures 3E, F**). There was no significant difference in the behavioral test between the normal group and sham group (P>0.05, **Figure 3**).

### Quantitative MRI Changes in the IVD and Muscles Tissue

**Figure 4:**
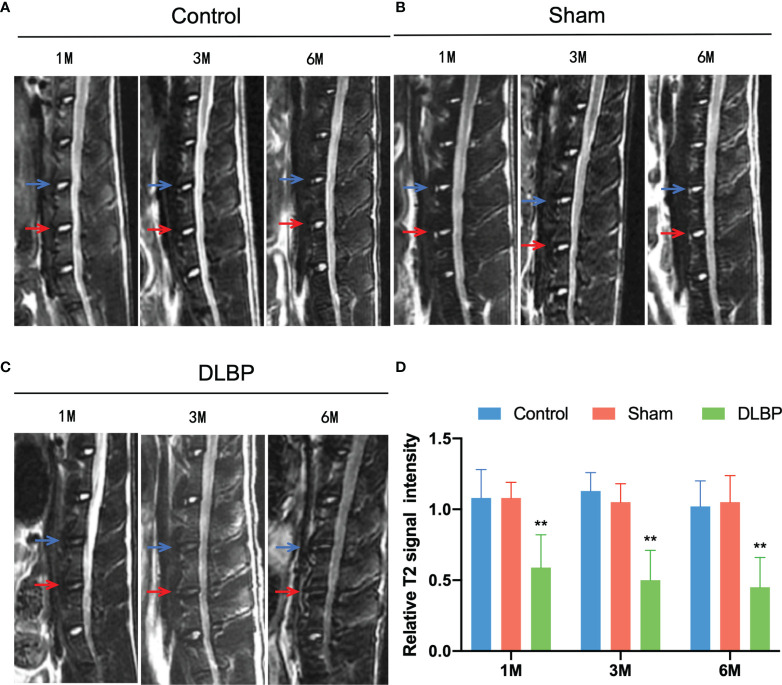
MRI T2WI of lumbar spine of rat models from 1 month and 6 months. **(A)** Control group, L4/5 IVD (blue) and L5/6 IVD (red); **(B)** Sham group; **(C)** DLBP group; **(D)** Bar chart of relative signal intensity of IVD among three groups. Compared with control group, **p < 0.01.

**Figure 5:**
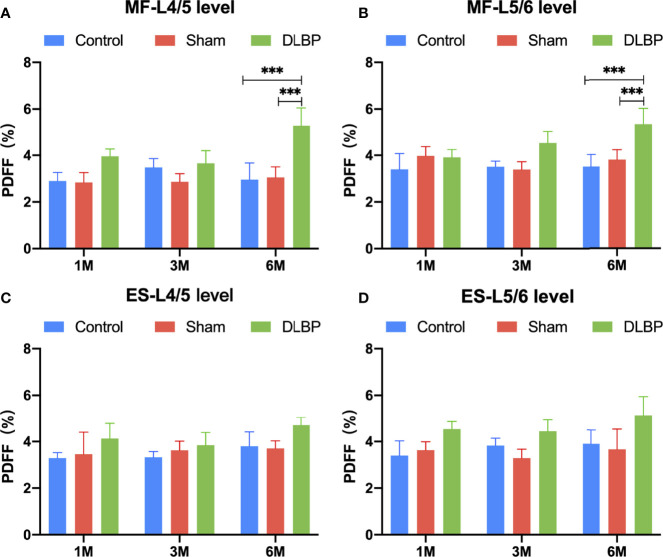
PDFF in paraspinal muscles at L4/5 and L5/6 levels from 1 month to 6 months. **(A)** PDFF of multifidus at L4/5 level; **(B)** PDFF of multifidus at L5/6 level; **(C)** PDFF of erector spinae at L4/5 level; **(D)** PDFF of erector spinae at L5/6 level. MF, multifidus; ES, erector spinae. Compared with control group and sham group, ***p < 0.001.

**Figure 6:**
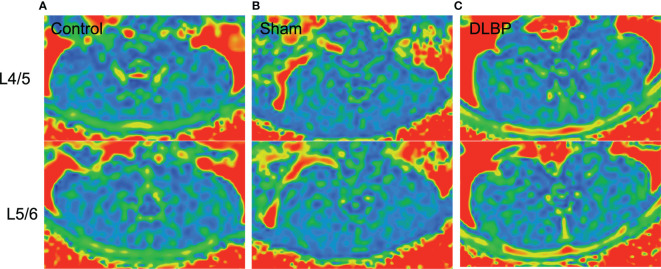
Representative MRI PDFF images of paraspinal muscles in DLBP rats. **(A)** Control group; **(B)** Sham group; **(C)** DLBP group.

1 month, 3 months and 6 months after lumbar IVDs puncture in rats, the relative signal intensity of DLBP were significantly lower than those in the normal group at the L4/5 and L5/6 (1M: 0.59 ± 0.23 *vs.*1.08 ± 0.20; 3M: 0.50 ± 0.21 *vs.* 1.13 ± 0.13;6M: 0.45 ± 0.21 *vs.*1.02 ± 0.18;P <0.05, **Figure 4**). The relative T2 signal intensity of the DLBP group in 3 months and 6 months was less than 1 month, but the difference was not statistically significant (P > 0.05, **Figure 4D**). At the levels of L4/5 and L5/6, the multifidus PDFF values of the DLBP group were more than those of the normal group and sham group in the 6 months, and the difference was statistically significant (P <0.05, **Figures 5**, **6**).

### Histologic and Structural Changes in IVD and Muscles Tissue and Increased TNF-α Expression

**Figure 7:**
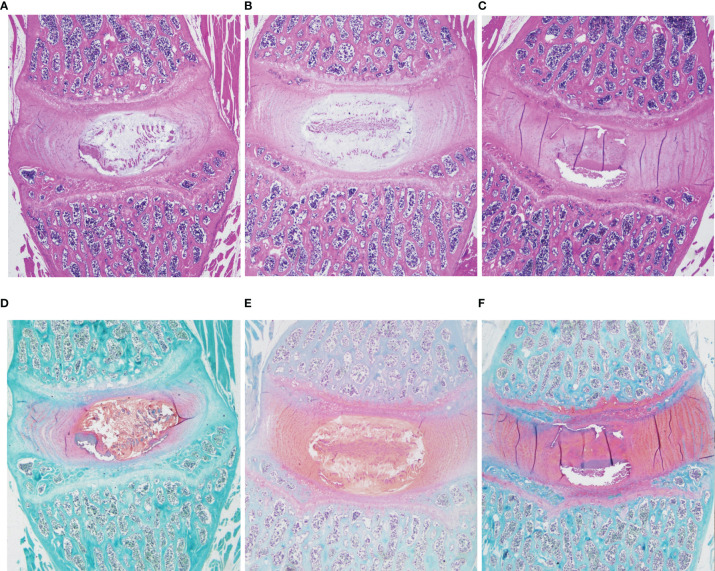
Representative histopathological images of IVD with HE and safranin O/fast green staining at 6 months in the control, sham and DLBP groups. **(A–C)** HE, ×20; **(D–F)** Safranin O/fast green staining, ×20. HE, hematoxylin and eosin.

**Figure 8:**
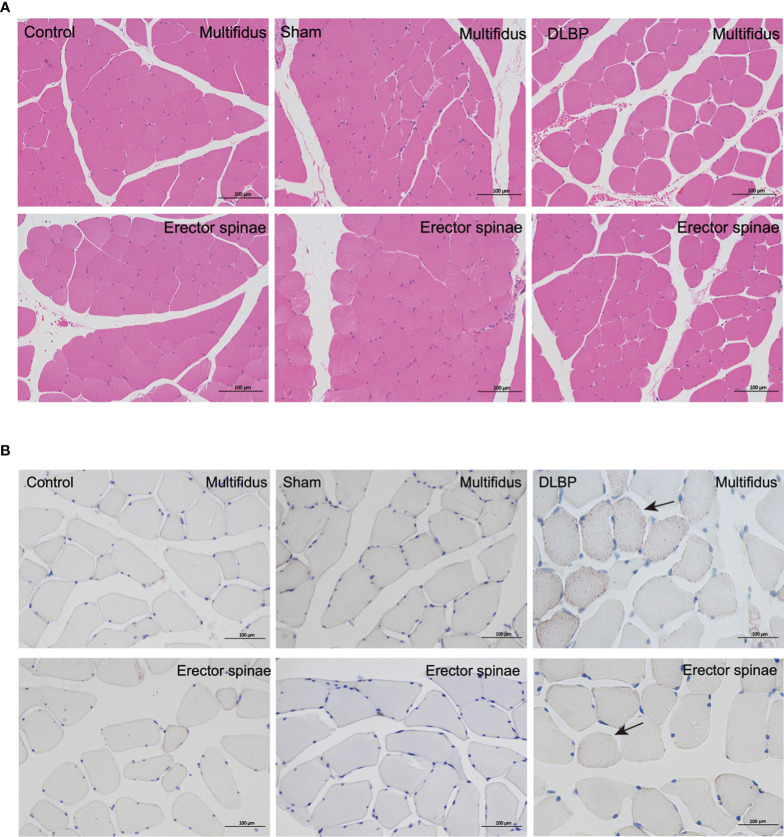
Representative histopathological images of muscles with HE and Oil Red staining at 6 months in the control, sham and DLBP groups. **(A)** HE, ×200; **(B)** Oil Red staining, ×200. lipid droplets (black arrow). HE, hematoxylin and eosin.

**Figure 9:**
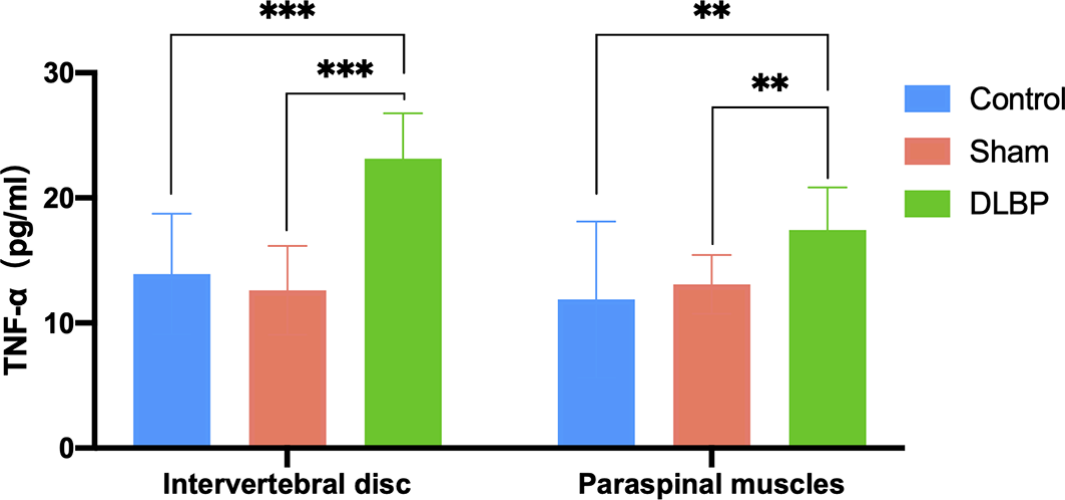
TNF-α levels in the IVD and paraspinal muscles at 6 months. Compared with control group and sham group, **p < 0.01, ***p < 0.001.

HE and Safranin Fast Green staining of IVDs showed that the histological morphology of the annulus fibrosus and nucleus pulposus in the DLBP group was changed. Compared with the control group and sham group, the collagen fibers of IVDs in the DLBP group were increased and twisted; the collagen in the cartilage was increased; the fibrous annulus was thickened, and the shape was distorted. The nucleus pulposus of the IVDs was replaced by hyperplastic cartilage cells (**Figure 7**). The HE and oil red O staining of the paraspinal muscles showed that the muscle bundle space of muscles in the DLBP group increased, and the lipid droplets in the muscle tissue increased (**Figure 8**). In addition, the expression of TNF-α in the IVDs and paraspinal muscles of the DLBP group were significantly higher than that of the control group and sham group (P <0.05, **Figure 9**). Compared with control and sham groups, TNF-α in multifidus was significantly increased in the DLBP group (DLBP *vs.* control *vs.* sham:19.52 ± 3.50 *vs.* 12.32 ± 6.65 *vs.* 12.71 ± 2.1, P <0.05). In the erector spinae, TNF-α of DLBP group were slightly increased, but no statistically significant differences were detected among three groups (DLBP *vs.* control *vs.* sham:15.33 ± 3.34 *vs.* 11.45 ± 5.96 *vs.* 13.47 ± 2.58, P >0.05).

## Discussion

In this study, we confirmed the increase in fat infiltration of the paraspinal muscles in clinical DLBP patients, which can be accurately quantified by MRI IDEAL-IQ. The IVDs were punctured under the guidance of X-ray fluoroscopy to establish a DLBP rat model. The animal model developed clinical symptoms, MRI findings and pathological changes consistent with human DLBP, which provides an experimental basis for studying DLBP IVDs and paraspinal muscles *in vivo*. In addition, we also found that the IVDs degeneration is directly related to the fat infiltration of the paraspinal muscles in rats and may be related to inflammation.

Several previous studies have shown that the fat infiltration of paraspinal muscles may be increased in chronic LBP (10, 32), but there has been controversy (33, 34). This is related to the semi-quantitative subjective method used to assess fat infiltration in previous studies. In this study, we attempted to apply more accurate quantitative MRI to determine whether the paraspinal muscles of DLBP patients have fatty infiltration. our cross-sectional clinical study showed a significant increase in PDFF of the multifidus and erector spinae muscles at the L4/5 and L5/6 levels in patients with DLBP. This may imply that fat infiltration of paraspinal muscles may be a strategy for the treatment of DLBP.

Based on the previously reported animal model of DLBP (19, 21, 35–37), we minimally puncture and inject PBS into the lumber IVDs under the guidance of X-ray fluoroscopy to establish a DLBP rat model suitable for observing muscle changes. In humans, L4/5 and L5/S1 are predilection sites of lumbar disc disease (38, 39). To gain a better DLBP animal model, the L4/5 and L5/6 discs were punctured in rat. So, the level of the punctured discs (L4/5 and L5/6) are preferentially observed in this study. Through animal behavior experiments, we observed that the rats in the DLBP group had obvious low back pain and muscle dysfunction. *In vivo*, rat lumbar spine MRI T2WI imaging showed that the relative signal intensity of L4/5 and L5/6 IVDs decreased progressively from 1 to 3 months after the puncture, which was consistent with the T2WI findings of patients with IVDs degeneration (40, 41). Ex vivo, the typical pathological changes of IVDs degeneration have been observed in the HE staining and Safranin Fast Green staining of rat IVDs, including shrinkage of nucleus pulposus, an increase of collagen fibers, and distortion and thickening of annulus fibrosus. These histological changes are similar to Suh’s DLBP model (21), but no obvious bone hyperplasia has been observed. This effective animal model is closer to the natural development of DLBP, because only the intervertebral disc is precisely damaged.

In experimental animals, we controlled degeneration of the lumbar IVDs as a single variable and then observe the dynamic development of the fat infiltration of the paraspinal muscle. After the rat’s IVDs were punctured, paraspinal muscles fat content was measured by quantitative MRI *in vivo*. We found that the PDFF of the multifidus muscle of DLBP rats was significantly increased compared to the control group, which is consistent with the results of humans. Six months after puncture, we observed the widening of the muscle bundle interval and intramuscular lipids in the HE staining and Oil Red O staining of the muscle.

However, the overall muscle PDFF is lower in DLBP rats than that of clinical patients, and the increase in erector spinae muscle is not significant. This may be related to the course of the disease duration and the more developed paraspinal muscles in rats (42). But this may also imply that the multifidus muscle is a more vulnerable muscle in the paraspinal muscles of DLBP. Our study found the causal relationship between IVD degeneration and paraspinal muscle fat in DLBP rats.

How the fat infiltration of paraspinal muscles occurs after IVD degeneration in the DLBP is unknown and complicated. Previous studies have reported the inflammatory response in degenerated IVDs, especially TNF-α (4, 43). In this study, the expression of TNF-α was simultaneously detected in the IVDs and paraspinal muscles of the DLBP rats. Recently, Zhu et al. found that inflammatory cells were increased in the paraspinal muscles in LBP by biopsy (44). Studies have revealed the regulatory relationship between inflammatory cells and muscle fat infiltration in diabetes (45, 46). But the interaction between inflammation in the IVD and inflammation in the muscle is still unclear. In an obesity mice model fed a high-fat diet, TNF-α may impair mitochondrial biogenesis and function in different tissues of obese rodents, suggesting that fat accumulation in skeletal muscle may be caused by movement dysfunction and inflammation (23). Meanwhile, impairment in white adipose tissue function, due to the abnormal fat accumulation, is characterized by increased production of specific pro-inflammatory proteins such as adipokines by white adipose tissue and of cytokines such as TNF by immune cells of the stromal compartment (24). In this study, inflammatory response may also contribute to the fat infiltration of paraspinal muscles in DLBP. Therefore, in this study, paraspinal muscles fat infiltration and inflammatory response may influence and promote each other in DLBP patients.

Our study has several limitations. First, due to the pelvis obscuring the L6/S1 intervertebral disc, we only punctured the L4-6 rat intervertebral disc. PBS was injected after puncturing the IVDs in this study. It may be more effective to inject some other drugs, such as nerve growth factor. Other chemicals may prove to be more effective in the future. We measured the total fat content of the paraspinal muscles in DLBP patients and rats, which will be valuable to further explore the fat distribution in the paraspinal muscles and clearly quantify the lipid levels inside and outside the muscle cells. Moreover, the regulatory relationship between inflammation and fat infiltration after IVDs degeneration is not yet clear, but we are actively conducting this research. Movement disorder after IVD puncture in rats is a potential influencing factor of fat infiltration of paraspinal muscles. Open-field test may be helpful in detecting the amount of exercise in rats. Finally, although the rat is the classic choice for the pain animal model, the physiology of humans and rats remains very different. We look forward to animal experiments closer to human species or high-quality clinical cohort studies to verify the role of inflammation in the fat infiltration of paraspinal muscles after intervertebral disc degeneration.

In conclusion, we confirmed that the paraspinal muscles have fat infiltration in the DLBP patients and rats, and that there is a causal relationship between fat infiltration and IVDs degeneration. In addition, we have developed and verified a novel rat model of DLBP. We believe that this model is more suitable for future studies of DLBP muscle-related pathological mechanisms and screening of treatment strategies. After IVDs degeneration, the paraspinal muscle fat infiltration and inflammation are closely related. The paraspinal muscle fat infiltration is the detection point of the therapeutic effect of DLBP and reducing the inflammation of the paraspinal muscle is a potential strategy for DLBP treatment.

## Data Availability Statement

The original contributions presented in the study are included in the article/supplementary material. Further inquiries can be directed to the corresponding author.

## Ethics Statement

The studies involving human participants were reviewed and approved by The Ethical Committee of Kunming Medical University. The patients/participants provided their written informed consent to participate in this study. The animal study was reviewed and approved by The Ethical Committee of Kunming Medical University. Written informed consent was obtained from the individual(s) for the publication of any potentially identifiable images or data included in this article.

## Author Contributions

BH: designed the study and conceived the report. YH and LW: wrote the draft of the manuscript and revised it critically. BL, KY, and YL: established the rat model and behavioral experiment. YH, XZ, and JC: human and animal MRI scan. YH, BL, KY, and ZZ: performed the histological experiment, including HE staining, Oil Red O staining and Safranin Fast Green Staining. YH, LW, XC and BH: data measurement, analysis and statistics. YH and LW: created the figures and tables. All authors contributed to the article and approved the submitted version.

## Funding

This work is supported by the Applied Basic Research Project of Yunnan Province- Kunming Medical University Joint Fund (202001AY070001-038, 202001AY070001-200), Yunnan Provincial Bone and Joint Disease Clinical Medicine Center Project (ZX2019-03-04), and Beijing Hospitals Authority Youth Programme (QMS20200402).

## Conflict of Interest

The authors declare that the research was conducted in the absence of any commercial or financial relationships that could be construed as a potential conflict of interest.

## Publisher’s Note

All claims expressed in this article are solely those of the authors and do not necessarily represent those of their affiliated organizations, or those of the publisher, the editors and the reviewers. Any product that may be evaluated in this article, or claim that may be made by its manufacturer, is not guaranteed or endorsed by the publisher.
